# Estimated birth weight and adult cardiovascular risk factors in a developing southern Chinese population: a cross sectional study

**DOI:** 10.1186/1471-2458-10-270

**Published:** 2010-05-24

**Authors:** CM Schooling, CQ Jiang, TH Lam, BJ Cowling, SL Au Yeung, WS Zhang, KK Cheng, GM Leung

**Affiliations:** 1 School of Public Health, Li Ka Shing Faculty of Medicine, The University of Hong Kong, Hong Kong SAR, China; 2 Guangzhou Number 12 Hospital, Guangzhou, China; 3 Department of Public Health and Epidemiology, University of Birmingham, Birmingham, UK

## Abstract

**Background:**

Birth weight is negatively associated with cardiovascular diseases and diabetes, but the associations are less well-established in developing populations where birth weight is often unavailable. We studied the association of birth weight and cardiovascular risk, using birth rank as an instrumental variable, in Southern China.

**Methods:**

We used published data on birth weight by birth rank from an appropriate population and baseline data from the Guangzhou Biobank Cohort Study phases 2 & 3 (2005-8) to examine the adjusted associations, using instrumental variable analysis, of birth weight with clinically measured cardiovascular risk factors and the metabolic syndrome in older (≥ 50 years) men (n = 5,051) and women (n = 13,907).

**Results:**

Estimated birth weight was associated with lower blood pressure (systolic -0.25 mm Hg 95% confidence interval (CI), -0.53 to 0.03 and diastolic -0.33 mm Hg 95% CI -0.48 to -0.18 per standard deviation higher birth weight), but had little association with glucose, lipids, waist-hip ratio, body mass index or the metabolic syndrome, adjusted for age, sex, early environment and number of offspring.

**Conclusion:**

Birth weight may impact blood pressure; however associations of birth weight with other cardiovascular risk factors may not be related to foetal exposures, but speculatively could be an historical co-incidence, with corresponding implications for prevention.

## Background

Lower birth weight is associated with higher risk of cardiovascular diseases (CVD) [1,2], hypertension [3], diabetes [4,5], and perhaps poor lipids in adulthood [6,7], although maternal diabetes in pregnancy and fetal over growth may also be implicated in diabetes [8]. Moreover some of the effects are fairly small, and the underlying causative exposure(s) and mechanism(s) driving the association between lower birth weight and adult cardiovascular diseases are not, as yet, fully understood, possibly because other aspects of fetal development, such as body composition at birth [9], are more important. Maternal adiposity may contribute to higher birth weight [10]. Perhaps because of the difficulty of manipulating birth weight [11], as well as potential ethical problems, almost all the evidence concerning humans comes from observational studies, which may be inherently open to uncontrollable biases. There are many experimental animal studies investigating the impact of pre-natal under-nutrition on CVD risk, whether these generalize to humans, where maternal supplementation has little effect on CVD risk [12], remains unclear [13], although the effects of over-nutrition may be clearer [14,15]. Moreover, most of these observations come from studies in long-term industrialised populations, where it is difficult to disentangle the effects of birth weight from its social context including previous inter-generational exposures and subsequent exposures across the life course, including growth and final size. In developing populations birth weight is typically lower than in long-term industrialised countries, and thus may be a relatively more important contributor to the growing epidemic of non-communicable chronic diseases, and hence also an important intervention target. However, there is increasing evidence that some associations between life course exposures and cardiovascular disease or its risk factors are epidemiological stage specific [16-18], perhaps because of epigenetic influences, making evidence from non-western settings valuable for developing effective interventions.

In developing populations birth weight, was not until recently, routinely measured and evidence concerning the association of birth weight with adult cardiovascular disease or its risk factors is mainly based on young people [18] or on small samples of inevitably atypical, hospital births with high attrition rates [18-22]. Given the long latency period following up a new cohort today could take decades to produce any evidence. In addition, associations between birth weight and cardiovascular disease or its risk factors may only be evident in adulthood or may amplify with age, making associations observed in childhood difficult to interpret.

Using birth rank as an instrumental variable for birth weight provides an alternative approach to observational studies for elucidating the contribution of birth weight to adult cardiovascular disease in general. Such an approach also provides a means of assessing the association in developing populations within a reasonable time frame. Birth rank has been observed to be consistently associated with birth weight in many different settings at various epidemiological stages [23-30]. On the other hand there is little reason to believe that birth rank is causally associated with cardiovascular disease or its risk factors [31,32]. To clarify the association of birth weight with cardiovascular disease risk factors in general and outside of long-term developed western settings, we used birth rank as an instrumental variable in a large study of older southern Chinese.

## Methods

### Sources of data

The Guangzhou Biobank Cohort Study is a collaboration amongst the Guangzhou No. 12 Hospital, the Universities of Hong Kong and Birmingham, and has been described in detail [33]. Recruitment of participants draws from "The Guangzhou Health and Happiness Association for the Respectable Elders", a community social and welfare association unofficially aligned with the municipal government where membership is open to anyone aged 50 years or older for a monthly, nominal fee of 4 Yuan (50 US cents). Recruitment for phase 2 took place from April 2005 to May 2006 and for phase 3 from September 2006 to January 2008. About 7% of permanent Guangzhou residents aged 50 years and over are members of "The Guangzhou Health and Happiness Association for the Respectable Elders", of whom 22% enrolled for phase 2 or 3 recruitment, constituting 65.9% of the total recruitment, and were included if they were capable of consenting, ambulatory, and not receiving treatment modalities which if omitted may result in immediate life threatening risk, such as chemotherapy or radiotherapy for cancer, or dialysis for renal failure. Participants underwent a half-day detailed medical interview, including disease history, and physical examination. In phases 2 and 3 the questionnaire was extended to include more questions concerning the participants' early life, including the question "How many children did your mother give birth to including you? And "How many of these were older than you"? The Guangzhou Medical Ethics Committee of the Chinese Medical Association approved the study and all participants gave written, informed consent before participation.

The detailed methods of measurement have been reported [33]. In brief, standing height was measured without shoes to the nearest 0.1 centimetre. Weight was measured in light clothing to the nearest 0.1 kilogram. Hip circumference was measured at the greatest circumference round the buttocks below the iliac crest. Waist circumference was measured horizontally around the smallest circumference between the ribs and iliac crest, or at the level of the naval for obese participants. Body mass index (BMI) was calculated as the weight in kilograms divided by the square of height in metres. Seated blood pressure was recorded as the average of the last two of three measurements, using the Omron 705CP sphygmomanometer. Fasting HDL-cholesterol, triglyceride, total cholesterol and glucose levels were determined by the Shimadzu CL-8000 Clinical Chemical Analyzer.

### Instrumental variable

**Figure 1:**
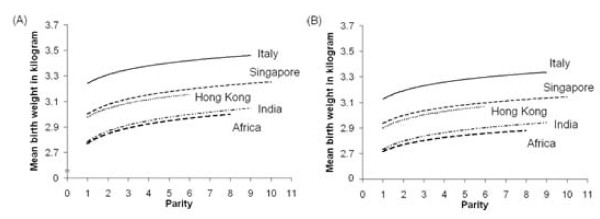
**Association between (A) male; (B) female birth weight and birth rank in the mid 20^th ^century from a variety of locations**[23-27].

The instrumental variable for birth weight was birth rank. We used published equations relating birth rank (of order 1 to 10+) to birth weight for 18,425 live born Chinese infants from poor families in Singapore from 1950 to 1951 [23]. This study represents 25% of all births in Singapore in the period, of which a summary is provided in Additional File 1 for easy reference. We used this source because it provides a large sample of Chinese from the relevant historic time. Moreover, the association between birth rank and birth weight is similar across populations, even though mean birth weight may differ. To illustrate this Figure 1 shows the association between birth rank and birth weight in the mid 20^th ^century from a variety of locations, including Singapore [23-27]. In addition, we also repeated our analysis using the association between birth rank and birth weight from all these studies combined. In Singapore in the mid 20^th ^century, the male infants were slightly heavier (mean birth weight 3.11 kilograms (kg), standard deviation (SD) 0.39) than the female infants (mean birth weight 3.03 kg, SD 0.37) [23].

### Outcome measures

We examined eight biological outcome measures, typically associated with cardiovascular disease: systolic blood pressure, diastolic blood pressure, fasting plasma glucose, HDL-cholesterol, total cholesterol, triglycerides, waist-hip ratio (WHR), and body mass index (BMI). We also examined the metabolic syndrome and its components using the International Diabetes Federation definition [34], with raised blood pressure as systolic blood pressure ≥ 130 mmHg or diastolic blood pressure ≥ 85 mmHg or treatment for hypertension, reduced HDL-C as HDL cholesterol <1.03 mmol/L in men or <1.29 mmol/L in women or treatment for this specific abnormality, raised fasting plasma glucose as fasting plasma glucose ≥ 5.6 mmol/L or previously diagnosed type 2 diabetes, raised triglycerides as triglycerides >1.7 mmol/L and central obesity as waist circumference ≥ 80 cm in women or ≥ 90 cm in men. The metabolic syndrome is the presence of central obesity and any two other components [34].

### Statistical analysis

We used a separate-sample instrumental variable (SSIV) estimator [35,36]. SSIV is similar to the more common two-stage least squares method, except that each stage is calculated using separate data. The first stage used birth rank to predict birth weight by sex from published data on birth weight in Chinese infants. The second stage used the regression predictions of birth weight from the first stage as independent variables predicting cardiovascular disease risk factors. From these models we reported adjusted mean differences (Δ) or odds ratios with 95% confidence intervals (CIs) per sex-specific standard deviation higher birth weight, so as to have a comparable measure in men and women. We also examined whether the associations with birth weight varied by age and sex from the p values of the relevant interaction terms. However, it is possible that birth rank is a better instrument among men than women, because the factors potentially associated with birth rank, such as childhood socioeconomic position, are not associated with CVD risk among men in this study [37], so we also presented sex-specific results.

Potential confounders considered were study phase, age (in 5-year age-groups), life course socio-economic position (parental possessions, education and job type), lifestyle habits (ever-smoking, use of alcohol and physical activity both leisure and other), number of offspring and measures of early life experience (leg length and seated height), categorized as in Table 1.We included potential confounders on a change in estimate criteria for blood pressure, on this basis we excluded job type and lifestyle habits. We should have excluded measures of early life experiences as well on this basis, however to ensure we had adjusted for environmental differences during growth we also included leg length and seated height in one of the models. Thus, we presented four models. Model 1 adjusted for use of appropriate medication (not included when analyzing metabolic syndrome or its components), study phase, age and sex. Model 2 additionally adjusted for parental possessions and education. Model 3 additionally adjusted for number of offspring, leg length and seated height. The role of adiposity is unclear, so Model 4 additionally adjusted for BMI and WHR, where appropriate.

**Table 1:** Characteristics by birth rank in 18,958 older Chinese in phases 2 and 3 of the Guangzhou Biobank Cohort Study (2005-8)

		Men				
		
		Birth rank				
		1	2	3	4+	p value
N		1,448	1,141	903	1,559	
Age (years)	Mean and (SD)	62.9 (7.0)	63.0 (7.0)	63.2 (7.0)	63.3 (7.0)	0.98
Height (cm)	Mean and (SD)	164.4 (6.2)	164.6 (5.8)	164.8 (5.8)	164.6 (5.9)	0.03
Leg length (cm)	Mean and (SD)	75.5 (4.2)	75.6 (3.8)	75.7 (4.0)	75.6 (3.9)	<0.001
Seated height (cm)	Mean and (SD)	88.9 (3.6)	88.9 (3.4)	89.0 (3.2)	88.9 (3.3)	0.003
						
Parental possessions	None	62.1	63.8	66.0	68.3	0.004
(Bicycle, watch	1 or 2	16.6	18.4	16.2	15.2	
and sewing machine)	All 3	19.6	16.3	16.6	14.4	
	Unknown	1.7	1.5	1.2	2.1	
						
Education	<Primary	3.0	2.1	2.3	2.3	0.89
	Primary	26.7	26.6	24.8	25.9	
	Junior middle	29.8	29.8	29.6	31.2	
	Senior middle	25.1	24.3	25.9	24.6	
	Junior college	9.3	9.8	10.1	10.1	
	College	6.0	7.4	7.3	6.0	
						
Job type‡	Manual	39.5	36.8	36.3	39.0	0.13
	Non-manual	29.5	34.7	33.2	32.0	
	Other	31.0	28.5	30.5	29.0	
						
Smoking status	Ever	61.9	61.3	61.5	61.1	0.97
						
Alcohol use	Never	46.4	48.6	51.0	46.6	0.20
	<1/week	28.1	28.1	27.3	30.3	
	1-4/week	6.6	7.8	6.5	6.5	
	5+/week	12.2	10.2	10.4	11.5	
	Ex-drinker	6.8	5.3	4.8	5.1	
						
Physical activity	Inactive	12.6	12.9	13.7	11.5	0.69
(IPAQ) †	Minimally active	47.7	46.5	48.1	47.7	
	HEPA active	39.8	40.6	38.2	40.8	
						
Offspring (number)	Mean and (SD)	2.3 (1.4)	2.3(1.4)	2.3 (1.3)	2.2 (1.3)	0.002
						
Cardiovascular risk factors	SBP (mmHg)	132.6 (21.0)	131.4 (20.8)	132.3 (21.6)	131.1 (21.2)	0.67
Mean and (SD) ¢	DBP (mmHg)	76.3 (11.3)	75.8 (11.4)	76.0 (11.1)	74.6 (10.8)	0.21
	FBG (mmol/L)	5.6 (1.7)	5.6 (1.7)	5.5 (1.3)	5.6 (1.5)	<0.001
	TG (mmol/L)	1.8 (1.4)	1.6 (1.2)	1.7 (1.4)	1.7 (1.4)	<0.001
	TC (mmol/L)	5.6 (1.1)	5.6 (1.0)	5.6 (1.1)	5.6 (1.1)	0.36
	HDL-C (mmol/L)	1.5 (0.4)	1.5 (0.4)	1.5 (0.4)	1.5 (0.4)	0.05
	WHR	0.9 (0.06)	0.9 (0.06)	0.9 (0.06)	0.9 (0.07)	0.01
	BMI (kg/m^2^)	23.6 (3.3)	23.5 (3.1)	23.4 (3.3)	23.5 (3.2)	0.03

		**Women**				
		
			**Birth rank**			
		**1**	**2**	**3**	**4+**	**p value**

N		3,961	3,074	2,375	4,497	
Age (years)	Mean and (SD)	59.8 (7.0)	59.4 (7.1)	59.1 (7.1)	59.1 (7.1)	0.84
Height (cm)	Mean and (SD)	154.1 (5.6)	154.2 (5.4)	154.2 (5.3)	153.9 (5.4)	0.03
Leg length (cm)	Mean and (SD)	70.2 (3.7)	70.1 (3.7)	70.2 (3.5)	70.0 (3.8)	0.002
Seated height (cm)	Mean and (SD)	83.9 (3.4)	84.0 (3.3)	84.0 (3.4)	84.0 (3.4)	0.47
						
Parental possessions	None	53.3	55.2	56.9	58.6	<0.001
(Bicycle, watch and sewing machine)	1 or 2	20.1	20.2	19.8	19.3	
	All 3	24.7	22.6	21.0	19.8	
	Unknown	1.9	2.0	2.3	2.4	
						
Education	<Primary	10.7	11.2	11.3	9.3	<0.001
	Primary	31.6	33.9	33.0	31.1	
	Junior middle	25.9	26.6	27.2	29.0	
	Senior middle	24.3	23.0	23.7	25.0	
	Junior college	5.9	4.1	3.4	4.4	
	College	1.6	1.3	1.5	1.3	
						
Job type‡	Manual	42.6	43.7	44.3	41.2	0.10
	Non-manual	27.0	27.9	26.5	28.2	
	Other	30.4	28.3	29.2	30.6	
						
Smoking status	Ever	3.2	2.7	2.3	2.3	0.04
						
Alcohol use	Never	73.5	73.5	73.6	72.2	0.12
	<1/week	20.5	20.9	21.7	21.2	
	1-4/week	1.6	1.8	1.5	1.8	
	5+/week	1.4	1.1	0.69	1.7	
	Ex-drinker	3.0	2.7	2.5	3.1	
						
Physical activity	Inactive	11.5	11.0	12.4	11.9	0.007
(IPAQ) †	Minimally active	44.3	42.2	42.0	40.3	
	HEPA active	44.2	46.8	45.6	47.9	
						
Offspring (number)	Mean and (SD)	2.3 (1.4)	2.2 (1.4)	2.2 (1.3)	2.1 (1.3)	0.29
						
Cardiovascular risk factors	SBP (mmHg)	127.2 (22.1)	127.2 (22.2)	126.9 (21.4)	125.9 (21.1)	0.005
Mean and (SD) ¢	DBP (mmHg)	72.0 (10.9)	72.1 (11.2)	72.0 (10.7)	71.2 (10.7)	0.02
	FBG (mmol/L)	5.6 (1.6)	5.6 (1.8)	5.6 (1.5)	5.6 (1.6)	<0.001
	TG (mmol/L)	1.7 (1.2)	1.7 (1.3)	1.7 (1.1)	1.7 (1.4)	<0.001
	TC (mmol/L)	6.1 (1.1)	6.1 (1.1)	6.1 (1.1)	6.1 (1.2)	0.17
	HDL-C (mmol/L)	1.7 (0.4)	1.7 (0.4)	1.7 (0.4)	1.7 (0.4)	0.18
	WHR	0.9 (0.07)	0.9 (0.06)	0.8 (0.07)	0.8 (0.07)	0.07
	BMI (kg/m^2^)	23.9 (3.4)	23.9 (3.4)	23.9 (3.4)	23.7 (3.3)	0.18

‡Manual occupations are agricultural worker, factory work or sales and service; non-manual are administrator/manager, professional/technical, military/disciplined

† health enhancing physical activity, i.e. vigorous activity at least 3 days a week achieving at least 1500 MET minutes per week on

activity on 7 days of the week achieving at least 3000 MET minutes per week

¢SBP: Systolic blood pressure; DBP: Diastolic blood pressure; FBG: Fasting blood glucose; TG: Triglycerides; TC: Total cholesterol; HDL-C: HDL-cholesterol; WHR: Waist hip ratio BMI: Body mass index

## Results

Of the 20,086 participants, 18,958 (94.4%) had complete data on all items of interest, so we did not perform imputations. There were more women (13,907) than men (5,051), and the women were younger (mean age 59.4 (SD 7.1)) than the men (mean age 63.1 (SD 7.0)). Age ranged from 50 to 96 years, but only 457 participants were older than 75 years.

Table 1 shows potential confounders and cardiovascular risk factors by birth rank, with birth ranks 4 to 10+ combined for compactness. Among men the instrumental variable, birth rank, had little association with potential confounders such as education, job type, smoking status, alcohol use and physical activity, although higher birth rank was associated with fewer parental possessions and fewer offspring. Birth rank was also associated with height, leg length and seated height. Among women birth rank was not associated with seated height, job type, alcohol use or number of offspring, although higher birth rank was associated with fewer parental possessions, lower education, manual job, never smoking and more physical activity.

Table 2 shows that birth weight was negatively associated with blood pressure, (diastolic and systolic) but had no association with glucose, triglycerides, total cholesterol, HDL-cholesterol, waist-hip ratio, body mass index in model 2. Adjustment for number of offspring, leg length and seated height, in model 3, attenuated the association between birth weight and systolic blood pressure. Adjustment for BMI and WHR, in model 4, did not change the estimates. There was little evidence that the association of birth weight and the outcomes concerned varied with sex, except for HDL-cholesterol, where there was a positive association among women, otherwise the pattern in men and women was similar. There was no evidence that the associations varied with age (data not shown).

**Table 2:** Instrumental variable estimate for the effect per birth weight standard deviation on cardiovascular disease risk factors in 18,958 older Chinese men and women in phases 2 and 3 of the Guangzhou Biobank Cohort Study (2005-8), overall and stratified by sex

				All		Men		Women		
				
¢Parameters	Men	Women	†Model	Δ	95%CI	Δ	95%CI	Δ	95%CI	¶p
SBP	131.8	126.7	1	-0.27	-0.55 to 0.009	-0.46	-1.01 to 0.08	-0.18	-0.50 to 0.15	0.41
(SD)	(21.1)	(21.7)	2	-0.29	-0.57 to -0.01	-0.46	-1.00 to 0.08	-0.23	-0.55 to 0.09	0.54
(mmHg)			3	-0.25	-0.53 to 0.03	-0.39	-0.93 to 0.15	-0.20	-0.52 to 0.12	0.59
			4	-0.24	-0.51 to 0.03	-0.45	-0.98 to 0.08	-0.17	-0.49 to 0.15	0.43
										
DBP	75.6	71.7	1	-0.35	-0.50 to -0.20	-0.54	-0.84 to -0.24	-0.28	-0.45 to -0.10	0.16
(SD)	(11.2)	(10.9)	2	-0.35	-0.50 to -0.20	-0.53	-0.83 to -0.23	-0.29	-0.47 to -0.12	0.21
(mmHg)			3	-0.33	-0.48 to -0.18	-0.51	-0.81 to -0.21	-0.27	-0.44 to -0.10	0.20
			4	-0.32	-0.47 to -0.18	-0.54	-0.83 to -0.26	-0.25	-0.42 to -0.08	0.11
										
FBG	5.6	5.6	1	0.007	-0.01 to 0.03	-0.02	-0.06 to 0.02	0.02	-0.007 to 0.04	0.10
(SD)	(1.6)	(1.6)	2	0.006	-0.01 to 0.03	-0.02	-0.06 to 0.02	0.02	-0.009 to 0.04	0.12
(mmol/L)			3	0.008	-0.01 to 0.03	-0.02	-0.06 to 0.02	0.02	-0.006 to 0.04	0.12
			4	0.009	-0.01 to 0.03	-0.02	-0.06 to 0.01	0.02	-0.004 to 0.04	0.08
										
TG	1.7	1.7	1	-0.008	-0.03 to 0.01	-0.03	-0.07 to 0.008	0.003	-0.02 to 0.02	0.10
(SD)	(1.4)	(1.3)	2	-0.007	-0.03 to 0.01	-0.03	-0.07 to 0.008	0.003	-0.02 to 0.02	0.11
(mmol/L)			3	-0.006	-0.02 to 0.01	-0.03	-0.07 to 0.007	0.004	-0.02 to 0.03	0.11
			4	-0.004	-0.02 to 0.01	-0.03	-0.07 to 0.003	0.007	-0.01 to 0.03	0.05
										
TC	5.6	6.1	1	0.01	-0.002 to 0.03	-0.005	-0.03 to 0.02	0.02	0.002 to 0.04	0.14
(SD)	(1.1)	(1.1)	2	0.01	-0.001 to 0.03	-0.005	-0.03 to 0.02	0.02	0.004 to 0.04	0.13
(mmol/L)			3	0.01	-0.003 to 0.03	-0.006	-0.04 to 0.02	0.02	0.002 to 0.04	0.14
			4	0.01	-0.003 to 0.03	-0.007	-0.04 to 0.02	0.02	0.002 to 0.04	0.12
										
HDL-C	1.5	1.7	1	0.006	-0.0002 to 0.01	-0.005	-0.02 to 0.006	0.009	0.002 to 0.02	0.03
(SD)	(0.4)	(0.4)	2	0.005	-0.0008 to 0.01	-0.005	-0.02 to 0.005	0.009	0.002 to 0.02	0.03
(mmol/L)			3	0.004	-0.002 to 0.01	-0.006	-0.02 to 0.005	0.008	0.001 to 0.01	0.03
			4	0.003	-0.002 to 0.009	-0.005	-0.01 to 0.005	0.006	-0.0005 to 0.01	0.07
										
WHR	0.9	0.8	1	-0.0004	-0.001 to 0.0005	0.0001	-0.002 to 0.002	-0.0006	-0.002 to 0.0005	0.49
(SD)	(0.06)	(0.07)	2	-0.0004	-0.001 to 0.0005	0.0002	-0.002 to 0.002	-0.0007	-0.002 to 0.0004	0.40
			3	-0.0002	-0.001 to 0.0007	0.0004	-0.001 to 0.002	-0.0004	-0.001 to 0.0006	0.38
										
BMI	23.5	23.8	1	-0.05	-0.09 to 0.002	0.008	-0.08 to 0.10	-0.06	-0.12 to -0.006	0.19
(SD)	(3.1)	(3.3)	2	-0.04	-0.09 to 0.007	0.01	-0.08 to 0.10	-0.06	-0.12 to -0.005	0.18
(kg/m^2^)			3	-0.04	-0.09 to 0.008	0.02	-0.07 to 0.10	-0.06	-0.12 to -0.005	0.15

†Model 1 adjusted for use of appropriate medication, study phase, age and where appropriate sex

Model 2 additionally adjusted for parental possessions and education

Model 3 additionally adjusted for number of offspring, leg length and seated height

Model 4 additionally adjusted for BMI and WHR

¢SBP: Systolic blood pressure; DBP: Diastolic blood pressure; FBG: Fasting blood glucose; TG: Triglycerides; TC: Total cholesterol; HDL-C: HDL-cholesterol; WHR: Waist hip ratio BMI: Body mass index

¶p value for interaction by sex

Table 3 shows that birth weight was negatively associated with the presence of raised blood pressure in all models but had no association with other components of the metabolic syndrome or the syndrome itself in any model. There was little evidence that the association of birth weight and metabolic syndrome or its components varied with sex, except for central obesity, where there were opposite associations by sex, although the confidence intervals included 1 for both men and women, otherwise the pattern in men and women was similar. There was no evidence that the associations varied with age (data not shown).

**Table 3:** Instrumental variable estimate (odds ratio) for the effect per birth weight standard deviation on cardiovascular disease risk factors in 18,958 older Chinese men and women in phases 2 and 3 of the Guangzhou Biobank Cohort Study (2005-8), overall and stratified by sex

	Proportion			All		Men		Women		
			
	Men	Women	†Model	Odds ratio	95%CI	Odds ratio	95%CI	Odds ratio	95%CI	¶p
Raised blood pressure	55.9%	47.2%	1	0.93	0.91 to 0.96	0.93	0.88 to 0.99	0.94	0.91 to 0.97	0.82
(≥ 130/85 mmHg or appropriate medication)			2	0.93	0.91 to 0.96	0.93	0.88 to 0.99	0.93	0.90 to 0.97	0.88
			3	0.94	0.91 to 0.96	0.93	0.88 to 0.99	0.94	0.90 to 0.97	0.88
			4	0.94	0.91 to 0.97	0.93	0.87 to 0.98	0.94	0.91 to 0.97	0.66
										
Reduced HDL-C	15.2%	19.3%	1	0.98	0.94 to 1.01	1.00	0.93 to 1.08	0.97	0.93 to 1.01	0.43
(<1.03 mmol/L for men; <1.29 mmol/L for women; or appropriate medication)			2	0.98	0.94 to 1.02	1.00	0.93 to 1.09	0.97	0.93 to 1.01	0.45
			3	0.98	0.94 to 1.02	1.00	0.93 to 1.08	0.97	0.93 to 1.02	0.47
			4	0.98	0.95 to 1.02	0.99	0.92 to 1.08	0.98	0.94 to 1.02	0.69
										
Raised fasting plasma glucose	33.7%	31.4%	1	1.02	0.99 to 1.05	1.04	0.98 to 1.10	1.01	0.98 to 1.05	0.49
(≥ 5.6 mmol/L or appropriate medication)			2	1.02	0.99 to 1.05	1.04	0.98 to 1.10	1.01	0.97 to 1.05	0.45
			3	1.02	0.99 to 1.05	1.03	0.97 to 1.10	1.01	0.98 to 1.05	0.44
			4	1.02	0.99 to 1.06	1.03	0.97 to 1.10	1.02	0.98 to 1.06	0.67
										
Raised triglycerides	34.2%	35.5%	1	0.99	0.96 to 1.02	0.97	0.91 to 1.03	1.00	0.97 to 1.04	0.27
(>1.7 mmol/L)			2	0.99	0.96 to 1.02	0.97	0.91 to 1.03	1.01	0.97 to 1.04	0.26
			3	1.00	0.97 to 1.03	0.97	0.91 to 1.03	1.01	0.97 to 1.04	0.26
			4	1.00	0.97 to 1.03	0.96	0.90 to 1.02	1.02	0.98 to 1.06	0.10
										
Central obesity	17.2%	34.9%	1	0.98	0.95 to 1.01	1.05	0.98 to 1.13	0.96	0.93 to 1.00	0.04
(waist circumference ≥ 80 cm for women or ≥ 90 cm for men)			2	0.98	0.95 to 1.01	1.05	0.98 to 1.13	0.96	0.93 to 1.00	0.03
			3	0.99	0.96 to 1.02	1.06	0.98 to 1.14	0.97	0.94 to 1.01	0.04
										
*Metabolic syndrome	11.6%	20.7%	1	0.98	0.94 to 1.02	1.04	0.95 to 1.13	0.97	0.93 to 1.01	0.15
			2	0.98	0.94 to 1.02	1.04	0.96 to 1.14	0.97	0.93 to 1.01	0.14
			3	0.99	0.95 to 1.03	1.05	0.96 to 1.14	0.97	0.93 to 1.02	0.15

†Model 1 adjusted for study phase, age and where appropriate sex

Model 2 additionally adjusted for parental possessions and education

Model 3 additionally adjusted for number of offspring, leg length and seated height

Model 4 additionally adjusted for BMI and WHR

¶p value for interaction by sex

*Metabolic syndrome defined according to the criteria of the International Diabetes Federation: Waist circumference ≥ 80 cm in women or ≥ 90 cm in men, plus any two of the following 4 factors: 1) triglyceride level > 1.7 mmol/L; 2) HDL cholesterol level <1.03 mmol/L in men or <1.29 mmol/L in women or treatment for this specific abnormality; 3) systolic blood pressure ≥ 130 mmHg or diastolic blood pressure ≥ 85 mmHg or treatment for hypertension; and 4) fasting plasma glucose level ≥ 5.6 mmol/L or previously diagnosed type 2 diabetes

Repeating the analysis using the association of birth rank with birth weight from five mid 20^th ^century studies produced similar results (Additional Files 2, 3).

## Discussion

In a large study of older people from an under-studied population using a novel approach we found that higher estimated birth weight was possibly associated with lower blood pressure and in women estimated birth weight was positively associated with HDL-cholesterol, but there was no association between birth weight and other lipids, fasting glucose or the metabolic syndrome. These findings are consistent with meta-analysis indicating that birth weight may have a small effect on blood pressure [3], although most publications focus only on systolic blood pressure [3]. These findings are also fairly consistent with studies suggesting that birth weight has little clear association with lipids [6,7], with mixed findings concerning sex-specific effects [38]. Our findings are less consistent with the association usually found between lower birth weight and diabetes [5]. However, this association is less clear outside of long-term developed western populations [18-20,39]. As such, our study extends observations concerning the long-term effects of birth weight in largely western populations to a developing population, with the advantage of using a potentially less confounded measure of pre-natal experience. Birth weight is usually socially patterned and positively associated with adult height [40], however birth rank had relatively little social patterning. There were significant differences in birth rank by parental possessions, because of our large sample size, but the absolute differences were small.

## Limitations

Despite using the instrumental variable approach to minimise confounding in a large sample in an under-studied population, there are several limitations. First, the infrastructure to facilitate fully representative cohort studies in developing countries such as China is not readily available, which could preclude evidence from a large proportion of the global developing population during a period of transition. Our findings would be biased if people with specific birth rank and cardiovascular risk factors were systematically excluded, such as first-borns with low blood pressure or later-borns with high blood pressure; we have little reason to believe this is likely. Second, survival bias is possible. If survivorship were an issue we would have expected differences in association between the older and younger age-groups, of which we found little evidence. Third, the assumptions for a valid instrument are that it should be associated with the exposure and only associated with the outcome via the exposure. It is possible that birth rank does not satisfy these assumptions in this population, despite the consistently observed association between birth rank and birth weight [23-29]. We could not verify the association between birth rank and birth weight in our cohort, because participants were largely unable to answer questions on their birth weight, probably because it had never been measured. In the current southern Chinese populations, birth rank is positively associated with birth weight [30]. The effect of birth rank on birth weight may be reduced by older brothers [41]. We did not ask about the siblings' sex; lack of this information would weaken our instrumental variable at random for which our large sample should compensate. It is also possible that birth rank may be an instrumental variable for infant growth, as well as or instead of birth weight, because infants with lower birth order often grow rapidly in the immediate post-natal period. Rapid post-natal growth is associated with cardiovascular disease risk factors [42]. We do not have infant growth rates for this cohort. However rapid infant growth is usually associated with several aspects of cardiovascular risk [42], not just blood pressure. As regards, the assumption concerning a lack of association between birth rank and potential confounders, this was more apparent among men than women. Moreover, childhood socio-economic position has little association with CVD risk among men in this population [37]. As such, birth rank may be a more suitable instrumental variable for men than women. However, the associations were similar in both sexes. We also had to use a separate sample instrumental variable approach, so we could not adjust for covariates in the prediction equation for birth weight nor could we test the strength of our instrument. We did carry out sensitivity analyses using prediction equations based on other studies and results were similar (Additional Files 2, 3).

Nevertheless, we cannot be sure of the strength of birth rank as an instrument, although we did find an association in the expected direction with blood pressure. It would be valuable, if birth rank were used an instrumental variable for birth weight in a single sample including birth rank, birth weight and cardiovascular risk factors, however we are not aware of any such analysis. Fourth, higher birth rank may be associated with poorer childhood conditions, due to a parental strategy of lower investment in each offspring, and thereby offset any benefits of higher birth weight. Parents could also pay greater attention to children of lower birth rank, however to what extent that might be relevant in a culture where children are traditionally cared for by their grandparents is difficult to assess. Moreover, there is little difference in height by birth rank (Table 1), height is not clearly associated with cardiovascular risk in this population [17], and we adjusted for several measures of early life environment (parental possessions, education, leg length and seated height). Finally, we are basing our conclusions on a set of largely null results, which could well be due to insufficiently sensitive methods, encompassing all stages of the study from fieldwork to analysis. However, we did find some of the expected associations between birth weight and cardiovascular risk, just not all of them.

Despite these limitations in our study, our findings suggest little association between birth weight and cardiovascular risk factors in a population with a recent history of economic development. Perhaps the relevant exposure(s) did not vary or foetal exposures impact blood pressure but other mechanisms, of which birth weight is a non-causal marker, underlie the commonly observed inverse association between birth weight and diabetes, such as genetic influences as in the 'foetal insulin hypothesis' [43]. Alternatively, more recent socio-biological conceptualisations of the changes in disease patterns with economic development [44] suggest that the commonly observed associations for diabetes could simply be an historical coincidence, generated by two unrelated secular trends that occur with economic development. Height increases over many generations of economic development [45], perhaps as epigenetic constraints on fetal, infant and childhood linear growth "wear off" [46,47]. Although trends in birth weight are less well documented, height determines birth weight [48]. Birth weight has certainly increased in southern Chinese over the last 60 years [25,30]. It is also becoming increasingly evident that sex-steroids also increase over generations of economic development [49-51], which predisposes women to breast cancer [52] and perhaps men to ischemic heart disease [37,53,54]. However, increasingly levels of sex-steroids over generations would also reduce vulnerability to diabetes because of the anabolic effects of sex-steroids on muscle mass [55], providing greater capacity for glucose disposal. The historical coincidence of these two separate secular trends would generate an apparent negative association between birth weight and diabetes, particularly in populations with a long history of economic development and hence heterogeneity in intra-population experience over generations. Conversely, in populations with a much more recent history of economic development and hence homogeneity of experience over generations there would be less association, as seen here and the cohorts from more recently developed populations [18-20].

## Conclusion

Although, our study is only preliminary and hypothesis generating, nevertheless it has some potential implications. It suggests that the developmental origins of health paradigm needs to be extended beyond birth weight and infancy to investigate how exposures across and within generations affect cardiovascular disease risk factors, and specifically to consider that the observed associations may be a historical co-incidence. It also suggests that there may not be a common foetal development pathway determining blood pressure, diabetes and lipids, consistent with the disparate trends in sub-types of cardiovascular disease with economic development. Moreover, if confirmed it suggests that in developing countries birth weight may not be a useful intervention target.

Rather than being determined by birth weight, some of the commonly observed associations between birth weight and cardiovascular diseases may be a marker of exposures across generations or even a historical co-incidence. Future studies of birth weight and cardiovascular risk should include measures of parental and/or grandparental exposures, so that the relative contribution of inter-generational exposures and birth weight can be examined. Moreover, comparisons of maternal exposures during pregnancy across ethnicities and epidemiological stages might help identify the underlying exposures driving associations between birth weight and cardiovascular disease risk. Meanwhile caution should be exercised in extrapolating observational studies from long-term developed populations to other settings.

## Competing interests

The authors declare that they have no competing interests.

## Authors' contributions

CMS originated the idea, designed this analysis, interpreted the results and wrote the first draft, with the help of GML, SLAY and BJC. SLAY revised the analysis and edited the manuscript. THL, KKC, CQJ designed the original study and acquired the data with the help of WSZ. All authors revised the manuscript critically for important intellectual content; and gave final approval of the version to be submitted.

## Pre-publication history

The pre-publication history for this paper can be accessed here:

http://www.biomedcentral.com/1471-2458/10/270/prepub

## Supplementary Material

Additional file 1 **Supplemental tables**. Published data concerning birth rank and birth weight extracted for easy reference from a report from Singapore in 1951/2[23] Table S1.1: Infant birth weight by birth rank and sex Table S1.2: Equation and coefficients to predict birth weight from birth rank. Click here for file

Additional file 2 Instrumental variable estimates for the effect per birth weight standard deviation on cardiovascular disease risk factors in 18,958 older Chinese men and women in phases 2 and 3 of the Guangzhou Biobank Cohort Study (2005-8), based on 5 mid 20^th ^century studies[23-27] of birth rank and birth weight. Click here for file

Additional file 3 Instrumental variable estimates (odds ratio) for the effect per birth weight standard deviation on cardiovascular disease risk factors in 18,958 older Chinese men and women in phases 2 and 3 of the Guangzhou Biobank Cohort Study (2005-8), based on 5 mid 20^th ^century studies[23-27] of birth rank and birth weight. Click here for file
