# Supplementary material for: Estimated birth weight and adult cardiovascular risk factors in a developing southern Chinese population: a cross sectional study
Source: BMC Public Health. 2010 May 24;10:270. doi: 10.1186/1471-2458-10-270 (PMC2887395; doi:10.1186/1471-2458-10-270)
Supplement: Additional file 1 — Supplemental tables. Published data concerning birth rank and birth weight extracted for easy reference from a report from Singapore in 1951/2[23] Table S1.1: Infant birth weight by birth rank and sex Table S1.2: Equation and coefficients to predict birth weight from birth rank. [file 1471-2458-10-270-S1.DOC]

Additional File 1

Published data concerning birth rank and birth weight extracted for easy reference from a report from Singapore in 1951/2 [23]

Table S1.1: Infant birth weight by birth rank and sex

|  | Male | | Female | |
| --- | --- | --- | --- | --- |
| Birth rank | Pounds: ounces | kilograms | Pounds: ounces | kilograms |
| 1 | 6:9.63 | 2.995 | 6:7.24 | 2.927 |
| 2 | 6:12.76 | 3.083 | 6:9.8 | 2.999 |
| 3 | 6:14 | 3.118 | 6:11.1 | 3.036 |
| 4 | 6:15.68 | 3.166 | 6:11.84 | 3.057 |
| 5 | 6:15.68 | 3.166 | 6:13.17 | 3.095 |
| 6 | 6:15.36 | 3.157 | 6:13.05 | 3.091 |
| 7 | 7:1.5 | 3.218 | 6:14.25 | 3.125 |
| 8 | 7:2.27 | 3.239 | 6:14.1 | 3.121 |
| 9 | 7:1.96 | 3.231 | 6:14.3 | 3.127 |
| 10+ | 7:3.3 | 3.269 | 6:14.34 | 3.128 |

Ounces converted to kilograms as one ounce equals to 0.028349 kilograms

Table S1.2: Equation and coefficients to predict birth weight from birth rank

| Equation | W=b_o_+b_1_P+b_2_P^2^  (W=predicted birth weight; P=birth rank) | | |
| --- | --- | --- | --- |
| Coefficient | Unit | Male | Female |
| b_0_ | Pounds: ounces | 6:8.355 | 6:5.886 |
|  | kilograms | 2.958 | 2.888 |
| b_1_ | ounces | 2.097 | 2.024 |
|  | kilograms | 0.059 | 0.057 |
| b_2_ | ounces | -0.110 | -0.123 |
|  | kilograms | -0.0031 | -0.0035 |

Ounces converted to kilograms as one ounce equals to 0.028349 kilograms
