# Supplementary material for: Estimated birth weight and adult cardiovascular risk factors in a developing southern Chinese population: a cross sectional study
Source: BMC Public Health. 2010 May 24;10:270. doi: 10.1186/1471-2458-10-270 (PMC2887395; doi:10.1186/1471-2458-10-270)
Supplement: Additional file 2 — Instrumental variable estimates for the effect per birth weight standard deviation on cardiovascular disease risk factors in 18,958 older Chinese men and women in phases 2 and 3 of the Guangzhou Biobank Cohort Study (2005-8), based on 5 mid 20th century studies[23-27] of birth rank and birth weight. [file 1471-2458-10-270-S2.DOC]

Additional File 2: Instrumental variable estimates for the effect per birth weight standard deviation on cardiovascular disease risk factors in 18,958 older Chinese men and women in phases 2 and 3 of the Guangzhou Biobank Cohort Study (2005-8), based on 5 mid 20^th^ century studies [23-27] of birth rank and birth weight.

| ¢Parameters | †Model | ∆ | 95%CI |
| --- | --- | --- | --- |
| SBP | 1 | -0.15 | -0.43 to 0.13 |
| (mmHg) | 2 | -0.20 | -0.47 to 0.08 |
|  | 3 | -0.15 | -0.43 to 0.13 |
|  | 4 | -0.15 | -0.42 to 0.12 |
|  |  |  |  |
| DBP | 1 | -0.25 | -0.40 to -0.10 |
| (mmHg) | 2 | -0.26 | -0.41 to -0.11 |
|  | 3 | -0.24 | -0.39 to -0.09 |
|  | 4 | -0.24 | -0.38 to -0.09 |
|  |  |  |  |
| FBG | 1 | 0.01 | -0.009 to 0.03 |
| (mmol/L) | 2 | 0.01 | -0.01 to 0.03 |
|  | 3 | 0.01 | -0.008 to 0.03 |
|  | 4 | 0.01 | -0.008 to 0.03 |
|  |  |  |  |
| TG | 1 | -0.01 | -0.03 to 0.007 |
| (mmol/L) | 2 | -0.01 | -0.03 to 0.007 |
|  | 3 | -0.01 | -0.03 to 0.009 |
|  | 4 | -0.009 | -0.03 to 0.009 |
|  |  |  |  |
| TC | 1 | 0.01 | -0.003 to 0.03 |
| (mmol/L) | 2 | 0.01 | -0.001 to 0.03 |
|  | 3 | 0.01 | -0.003 to 0.03 |
|  | 4 | 0.01 | -0.003 to 0.03 |
|  |  |  |  |
| HDL-C | 1 | 0.005 | -0.001 to 0.01 |
| (mmol/L) | 2 | 0.004 | -0.002 to 0.01 |
|  | 3 | 0.003 | -0.002 to 0.009 |
|  | 4 | 0.003 | -0.003 to 0.008 |
|  |  |  |  |
| WHR | 1 | -0.0002 | -0.001 to 0.0007 |
|  | 2 | -0.0002 | -0.001 to 0.0007 |
|  | 3 | 0.00 | -0.0009 to 0.0009 |
|  |  |  |  |
| BMI | 1 | -0.04 | -0.09 to 0.007 |
| (kg/m^2^) | 2 | -0.04 | -0.09 to 0.01 |
|  | 3 | -0.04 | -0.08 to 0.01 |

†Model 1 adjusted for study phase, age and sex

Model 2 additionally adjusted for parental possessions and education

Model 3 additionally adjusted for number of offspring, leg length and seated height

Model 4 additionally adjusted for BMI and WHR

¢SBP: Systolic blood pressure; DBP: Diastolic blood pressure; FBG: Fasting blood glucose; TG: Triglycerides; TC: Total cholesterol; HDL-C: HDL-cholesterol; WHR: Waist hip ratio BMI: Body mass index
