# Supplementary material for: Estimated birth weight and adult cardiovascular risk factors in a developing southern Chinese population: a cross sectional study
Source: BMC Public Health. 2010 May 24;10:270. doi: 10.1186/1471-2458-10-270 (PMC2887395; doi:10.1186/1471-2458-10-270)
Supplement: Additional file 3 — Instrumental variable estimates (odds ratio) for the effect per birth weight standard deviation on cardiovascular disease risk factors in 18,958 older Chinese men and women in phases 2 and 3 of the Guangzhou Biobank Cohort Study (2005-8), based on 5 mid 20th century studies[23-27] of birth rank and birth weight. [file 1471-2458-10-270-S3.DOC]

Additional File 3: Instrumental variable estimates (odds ratio) for the effect per birth weight standard deviation on cardiovascular disease risk factors in 18,958 older Chinese men and women in phases 2 and 3 of the Guangzhou Biobank Cohort Study (2005-8), based on 5 mid 20^th^ century studies [23-27] of birth rank and birth weight.

|  | †Model | Odds ratio | 95% CI |
| --- | --- | --- | --- |
| Raised blood pressure | 1 | 0.94 | 0.92 to 0.97 |
| (≥130/85mmHg or | 2 | 0.94 | 0.92 to 0.97 |
| Having medication) | 3 | 0.94 | 0.92 to 0.97 |
|  | 4 | 0.95 | 0.92 to 0.97 |
|  |  |  |  |
| Reduced HDL-C | 1 | 0.97 | 0.93 to 1.00 |
| (<1.03mmol/L for men; | 2 | 0.97 | 0.93 to 1.00 |
| <1.29mmol/L for women; | 3 | 0.97 | 0.93 to 1.01 |
| Having medication) | 4 | 0.97 | 0.93 to 1.01 |
|  |  |  |  |
| Raised fasting plasma glucose | 1 | 1.03 | 0.99 to 1.06 |
| (≥5.6mmol/L or having | 2 | 1.02 | 0.99 to 1.06 |
| medication) | 3 | 1.02 | 0.99 to 1.06 |
|  | 4 | 1.03 | 1.00 to 1.06 |
|  |  |  |  |
| Raised triglycerides | 1 | 0.99 | 0.96 to 1.02 |
| (>1.7mmol/L) | 2 | 0.99 | 0.97 to 1.02 |
|  | 3 | 1.00 | 0.97 to 1.03 |
|  | 4 | 1.00 | 0.97 to 1.03 |
|  |  |  |  |
| Central obesity | 1 | 0.98 | 0.95 to 1.01 |
| (waist circumference≥80cm | 2 | 0.98 | 0.95 to 1.01 |
| for women or ≥90cm for men) | 3 | 0.98 | 0.95 to 1.02 |
|  |  |  |  |
| *Metabolic syndrome | 1 | 0.99 | 0.95 to 1.03 |
|  | 2 | 0.99 | 0.95 to 1.03 |
|  | 3 | 1.00 | 0.96 to 1.03 |

†Model 1 adjusted for study phase, age and sex

Model 2 additionally adjusted for parental possessions and education

Model 3 additionally adjusted for number of offspring, leg length and seated height

Model 4 additionally adjusted for BMI and WHR

*Metabolic syndrome defined according to the criteria of the International Diabetes Federation: Waist circumference ≥80cm in women or ≥90cm in men, plus any two of the following 4 factors: 1) triglyceride level>1.7mmol/L; 2) HDL cholesterol level <1.03 mmol/L in men or <1.29mmol/L in women or treatment for this specific abnormality; 3) systolic blood pressure≥130mmHg or diastolic blood pressure≥85mmHg or treatment for hypertension; and 4) fasting plasma glucose level ≥5.6mmol/L or previously diagnosed type 2 diabetes
